# Large-Area Fabrication of Vertical Silicon Nanotube Arrays *via* Toroidal Micelle Self-Assembly

**DOI:** 10.1021/acs.langmuir.0c03431

**Published:** 2021-01-28

**Authors:** Nadezda Prochukhan, Andrew Selkirk, Ross Lundy, Elsa C. Giraud, Tandra Ghoshal, Clive Downing, Michael A. Morris

**Affiliations:** † School of Chemistry, CRANN and AMBER Research Centres, Trinity College Dublin, College Green, Dublin 2, Ireland; ‡ BiOrbic—Bioeconomy SFI Research Centre, University College Dublin, Belfield, Dublin 4, Ireland

## Abstract

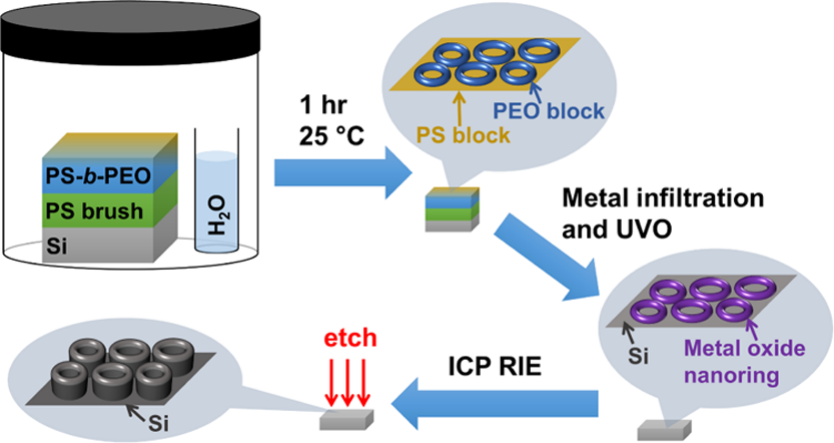

We present a highly scalable, room-temperature strategy for fabricating vertical silicon nanotube arrays derived from a toroidal micelle pattern *via* a water vapor-induced block copolymer (BCP) self-assembly mechanism. A polystyrene-*b*-poly(ethylene oxide) (PS-*b*-PEO) BCP system can be self-assembled into toroidal micelle structures (diameter: 400–600 nm) on a PS-OH-modified substrate in a facile manner contrasting with other complex processes described in the literature. It was found that a minimum PS-*b*-PEO thickness of ∼86 nm is required for the toroidal self-assembly. Furthermore, a water vapor annealing treatment at room conditions (∼25 °C, 60 min) is shown to vastly enhance the ordering of micellar structures. A liquid-phase infiltration process was used to generate arrays of iron and nickel oxide nanorings. These oxide structures were used as templates for pattern transfer into the underlying silicon substrate *via* plasma etching, resulting in large-area 3D silicon nanotube arrays. The overall simplicity of this technique, as well as the wide potential versatility of the resulting metal structures, proves that such room-temperature synthesis routes are a viable pathway for complex nanostructure fabrication, with potential applicability in fields such as optics or catalysis.

## Introduction

Block copolymer (BCP) self-assembly provides an avenue for the formation of a myriad of nano-scale morphologies, with applications such as optoelectronics, biosensing, filtration, bioactive surfaces, surface coatings, and magnetic applications, among others.^1−10^ More usual microphase separated self-assembled architectures include lamellar, cylindrical, spherical, and gyroidal structures as well as various micellar-based morphologies such as helices, tubes, disks, and toroids.^11−13^ Micelle formation is defined as the self-assembly of an amphiphilic BCP in a solvent medium to form a structure typically with a core and corona.^11,14^ Of the myriad of available micellar structures, toroidal micelles, in particular, have garnered interest owing to their proven applicability in synthesizing unique nanostructured materials with unique plasmonic and magnetic properties.^15,16^ Such structures are typically fabricated *via* incorporating various metals and metal oxides, which upon removal of the polymer matrix produce metallic toroidal or nanoring structures.^17^ These metal/metal oxide structures have also been utilized as hard masks for patterning underlying silicon substrates, yielding high aspect ratio nanotube arrays.^18,19^ Nonetheless, one notable drawback of toroidal micelles obtained *via* BCP or triblock copolymer self-assembly is that the size distributions of the structure are often too small for optoelectronic applications.^13^

In this work, we describe a novel strategy to address this issue by controlling the formation of large toroidal structures *via* water vapor annealing (WVA) of a polystyrene-*b*-poly(ethylene oxide) (PS-*b*-PEO) diblock copolymer system atop a PS-modified substrate. Vapor annealing is a common technique employed to direct BCP self-assembly *via* polymer swelling.^20,21^ Solvent vapor can selectively swell one or more blocks (thereby affecting the morphology) and the propensity of swelling can be estimated by the Flory–Huggins interaction parameter χ.^18,21^ The solvent vapor-driven swelling creates free volume, increasing the chain mobility.^18^ This is also described as plasticization and results in effective reduction of the glass-transition temperature and effective χ parameter.^22^ Thus, annealing times can be significantly reduced for high χ systems.^21^ Previously, WVA treatment has been employed to modify silk material or perovskite structures;^23−26^ however, water was only used as a facilitating solvent during BCP annealing rather than a unique swelling solvent.^27,28^

Furthermore, aside from the influence of the affinity of each block to the annealing solvent, the substrate effects can also substantially impact the resulting BCP pattern.^29−31^ The application of a polymer brush enables the tailoring of the surface energy (SE) of the substrate, influencing the BCP morphology.^32^ In this case, a PS brush is utilized to control the self-assembly of a lamellar PS-*b*-PEO system. The pre-treatment of the Si substrate with a PS brush favors PS block segregation to the brush/BCP interface.^29^ Qiu *et al.* demonstrated a similar approach to generate toroidal micelles in solution whereby a homopolymer was added to a BCP solution in various mass ratios in order to control the size of the micelle.^33^ The addition of a homopolymer to a BCP system requires careful tailoring; in addition to varying the micelle sizes, it can also result in the alteration of the shapes of the resulting micelles.^11,33−35^ Previous studies on toroidal micelle fabrication use highly acidic conditions to enforce ring micelle formation at surfaces or variation of solvent composition of BCP in solution^13,15^ and/or homopolymer addition to BCP solutions.^33^ Contrastingly, in this methodology we achieve such structures *via* simple surface modification.

The general mechanism by which toroid structures form from spherical or cylindrical micelles is described by Cui *et al.*, whereby high interfacial curvature of spheres or high-energy endcaps of cylinders result in transition to more stable toroid systems with lower free energy.^36^ In binary systems, toroids are generally not observed as the statistical probability of toroid formation is lower than the growth of rods as explained by Porte.^37^ The reason is simply that open shapes like rods have considerably more bent conformations possible than closed rings even though rings should be more energetically favorable.^37^ Therefore, in this case, toroid formation in a BCP system only occurs when polymer blocks are sufficiently flexible and self-attraction between blocks is considerable to form toroids from cylinders,^35−37^ such as is the case for PEO.^38^

Our process establishes that a homopolymer pre-treatment of the substrate can influence the onset of toroidal micellization of the BCPs within minutes of BCP deposition (<5 min). We use the resulting morphology to demonstrate a simple, yet original strategy for production of vertical silicon nanotube arrays (diameter = 355 ± 73 nm, height after 2 min etch = 355 ± 36 nm) *via* WVA (∼60 min), metal ion inclusion, and inductively coupled plasma (ICP) reactive ion etch (RIE). We use the term array to describe a densely packed arrangement of structures with little periodicity as no single self-assembled entities are observed. Traditional methods for silicon nanotube growth often include extensive chemical steps or require toxic chemicals such as silanes, acidic conditions, and high processing temperatures.^39−41^ Therefore, our methods are relatively facile compared to existent methods of production of metallic nanorings and vertical silicon nanotube vertical arrays, which usually involve complicated multistep synthesis and top-down approaches such as wet chemistry synthesis, chemical vapor deposition, various lithographic approaches, and use of multiple resist layers and materials.^19,42−46^ Additionally, WVA treatment is a low-cost and environmentally friendly fabrication method. One of the critical advantages of our approach is the formation of large feature size patterns by advantaging a highly controlled micelle approach to compliment smaller feature sizes produced by microphase separation of the systems. Morphology formation is simple and scalable, generating toroidal nanostructure array short timescales. We expect that the versatility of this patterning approach can be transferred to a range of PS-treated substrates to produce metallic nanorings or etched structures.

## Results and Discussion

### Pattern Formation

**Scheme 1:**
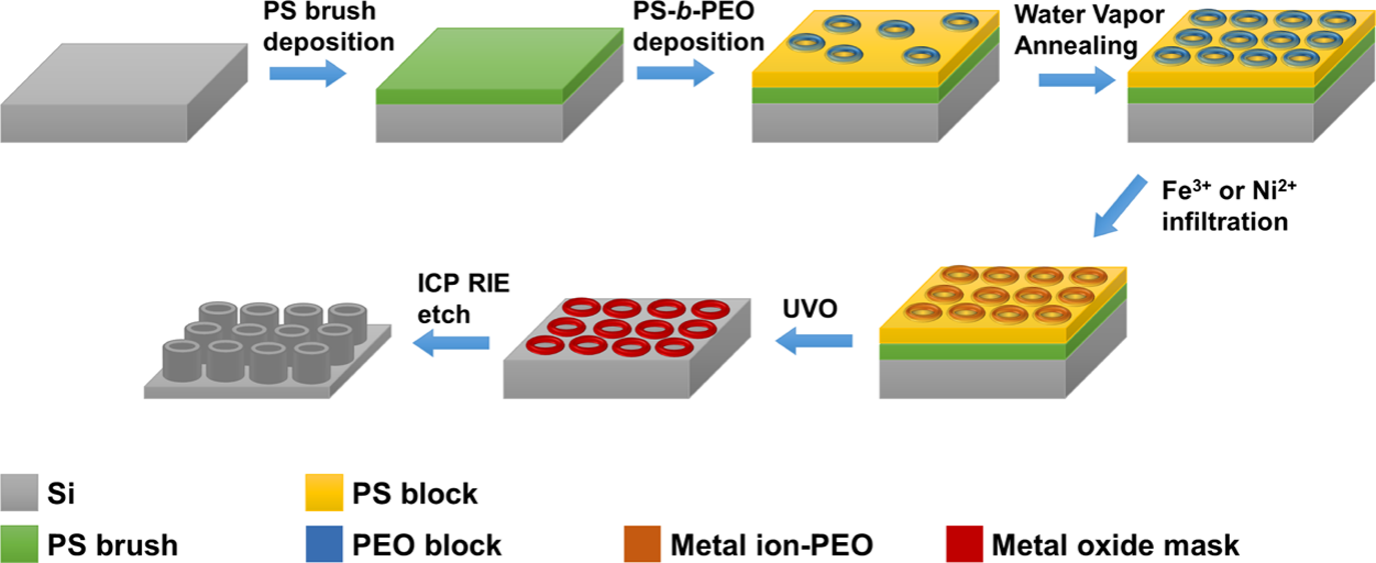
Toroidal Micelle Formation Process and Etch Pattern Formation

We chose a lamellar PS-*b*-PEO (24–24.5k) system in order to investigate whether surface pre-treatment and WVA can influence the resultant film morphology. The overall experiment is outlined in Scheme 1. In summary, we spin-cast PS-*b*-PEO/toluene solutions onto the PS pre-treated substrates. Subsequently, a WVA step was employed for an hour at 25 °C, which was followed by metal ion liquid phase infiltration and pattern transfer.

**Figure 1:**
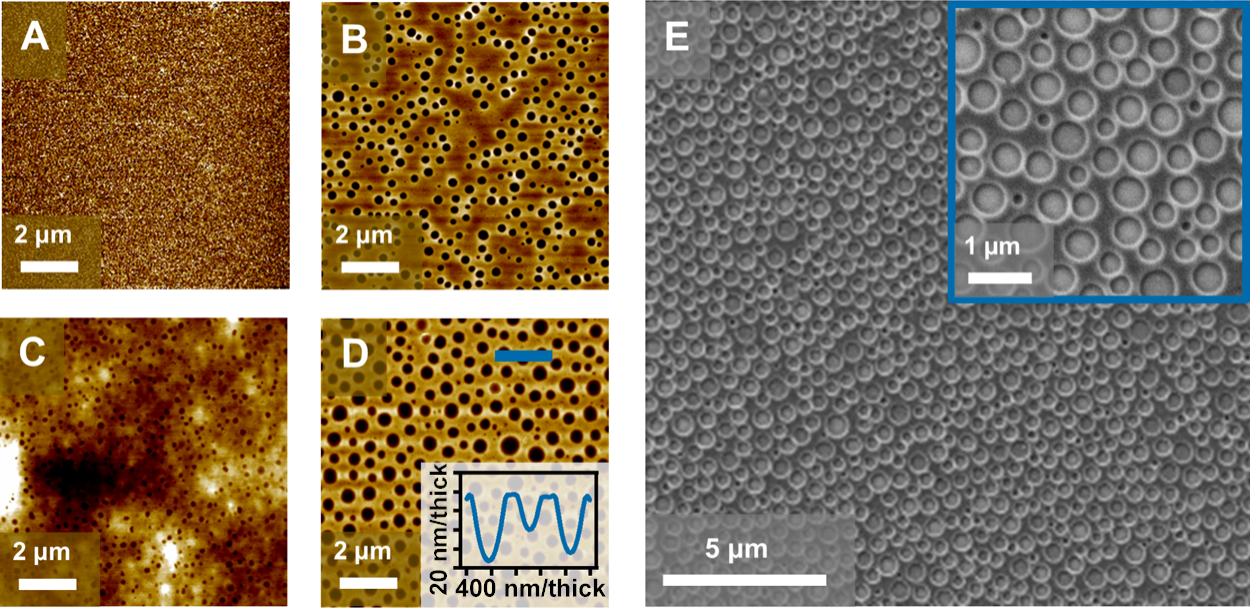
AFM images of (A) 10k PS brush optimized according to Lundy *et al.*,^48,49^ (B) 2 wt % PS-*b*-PEO on a brush as spun on without annealing shows disordered micelle formation after 1–5 min after spin-coating, (C) 2 wt % PS-*b*-PEO on Si after WVA, (D) 2 wt % PS-*b*-PEO on a PS brush after WVA. (E) Large-area SEM image of 2 wt % PS-*b*-PEO on a PS brush after WVA with a magnified image inset.

We observed the formation of a toroidal micelle-like pattern where PEO forms a circular micelle on a PS brush surface as seen from Figure 1B,D,E. This approximates to previous work by Wang *et al.*, where a toroidal pattern was formed using a BCP system with relatively similar volume fractions of the block.^47^ However, these workers used an ethanol-induced reconstruction to produce open micelle structures or toroidal structures from an initial spherical micelle array.

The attachment of a brush shown in Figure 1A is critical in tuning the SE such that micellar toroid-like surface features are obtained rather than microphase-separated structures.^15^ Spin-coating onto a PS brush immediately leads to the formation of micelles without the need of annealing, signifying the importance of the PS pre-treatment for the formation of micelle patterns (Figure 1B).

The toroidal pattern formation was promoted by selective swelling of the hydrophilic PEO block *via* exposure to water vapor. It is important to note that spin-coating the 2 wt % PS-*b*-PEO BCP solutions directly onto silicon substrates (Si) yields disordered micelles upon WVA (Figure 1C). Thus, effectively the PS brush promotes toroid micelle formation by matching SE and the availability of the brush increases the PS ration or the core volume of the micelle as expected, with the PEO block forming a corona around the PS core.^33,34^

Figure 1D,E displays the morphology of the BCP films on PS substrates after WVA. The PS-*b*-PEO film thickness (including the PS brush) is approx. 86 ± 1 nm for a water-annealed film as measured by reflectometry and the average “pore” or feature size is 364 ± 101 nm. Annealing films with water for less than an hour provides poor pattern homogeneity, whereas times of 60 min or longer result in a more evenly distributed BCP pattern. WVA considerably improves the toroid pattern packing, but the question is what mechanisms are at play.

To explain WVA in a quantitative way, we calculated the Flory–Huggins χ parameters for solvent–polymer interactions using the Hansen approach^50^
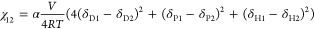
1where indices 1 and 2 represent the two components, that is, polymer and solvent; *V* is the molar volume of solution, α is a constant and assumed to be unity for systems whereby dispersive forces are dominant, *R* is the ideal gas constant, *T* represents the temperature; δ_D_, δ_P_, and δ_H_ are the dispersion, polar, and hydrogen-bonding interactions, respectively.^50,51^ The solvent and polymer are completely miscible if χ_12_ ≤ 0.5 as described by the Flory–Huggins model.^50,51^ Water is not theoretically selective for either of the blocks, that is, χ > 1. PEO miscibility with water is largely underestimated by the Flory–Huggins parameter; however, it is more important to consider the difference in polymer–solvent interaction parameters between the PS and PEO blocks. χ_S–H_2_O_ = 3.11 and χ_EO–H_2_O_ = 1.84 as estimated from eq 1 at 25 °C and with appropriate solubility parameters.^51^ We can thus postulate that the PEO block is responsible for the majority of microphase separation during WVA as well as for the formation of micelles. Additionally, the extremely low solubility of the PS block in water is another significant factor. It means that introducing minimal amounts of water to the BCP system would result in substantial increase in the interfacial free energy, thereby promoting micellization as observed.^14^

**Figure 2:**
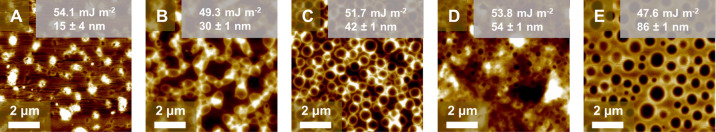
AFM images of PS-*b*-PEO on a PS brush after WVA cast from different concentration polymer solutions with associated SE and thickness (measured by reflectometry). Morphology of the films produced from (A) 0.2, (B) 0.5, (C) 0.8, (D) 1, and (E) 2 wt % polymer solutions after WVA.

We also looked at film thickness and SE effects on the observed morphology in order to investigate the onset of toroid formation as summarized in Figure 2. The films produced from 0.2 and 0.5 wt % solutions (Figure 2A,B) display no evident micellar pattern. The 0.8 wt % water-annealed samples form a similar structure to 2 wt % samples as seen in Figure 2C. However, the 0.8 wt % samples do not display a nanoring morphology after metal infiltration and UV/ozone (UVO) treatment (Supporting Information, Figure S2). Figure 2D also shows no observable ordered pattern for a 1 wt % film. Only upon reaching the thickness of 86 ± 1 nm, toroidal micelle formation becomes evident as observed for a 2 wt % sample in Figure 2E. We can thus deduce that a minimal thickness of approx. 86 nm is required for micelle evolution in this system. The results reveal that the morphology of micelles in thin films depends on the thickness of the thin films and the selectivity of the confining surfaces. It is well-known that film thickness can affect the resulting micelle morphology.^52^ To complement these findings, we estimated the SE of the films to try to understand the relative block composition at the surface. The SE was measured from advancing contact angles (CAs) with diiodomethane, water, and glycerol as test liquids and the calculation is outlined in the Supporting Information, Section S2. The 2 wt % water-annealed films display an SE of 47.6 mJ m^–2^. This is comparably higher than the PS pellet SE of 42.5 mJ m^–2^ but lower than the SE displayed by the poly(ethylene glycol) (PEG) melt of 54.8 mJ m^–2^, indicating the presence of both blocks at the surface.

### Metal Ion Inclusion

These self-assembled toroidal structures offer an ideal template to facilitate the formation of a Si nanotube array using a well-developed BCP lithographic strategy of liquid-phase selective infiltration and using a resultant metal oxide pattern as a mask in an etch process.^18,53^ The PEO domain readily coordinates various metals such as iron or nickel ions, which after processing in turn act as a hard mask for ICP etching.^53,54^

**Figure 3:**
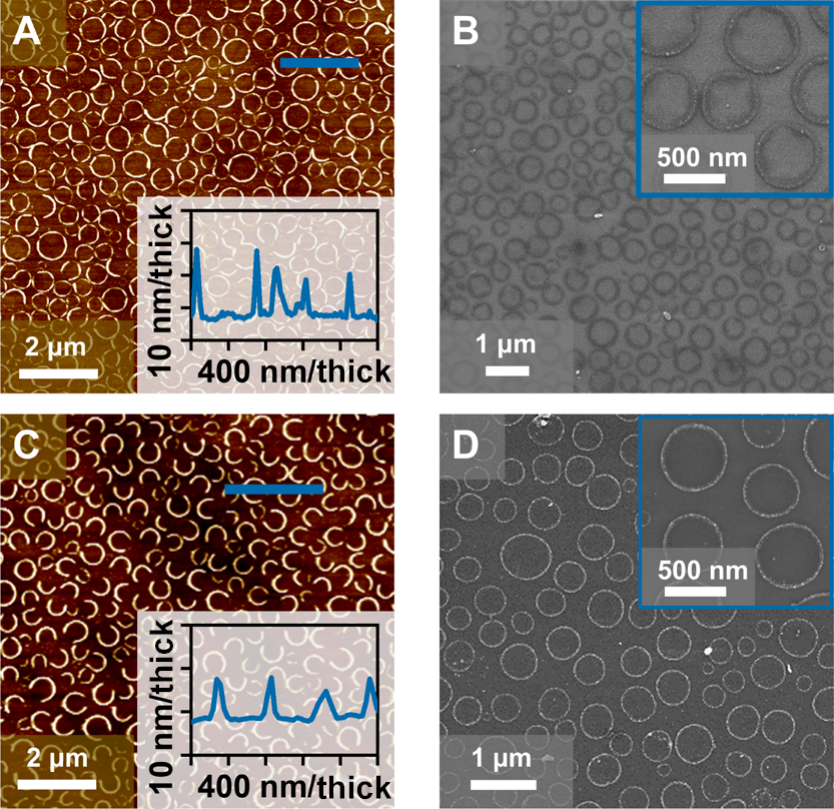
(A,B) Iron oxide mask AFM and SEM micrographs, respectively. (C,D) Nickel oxide mask AFM and SEM images, respectively.

Following WVA, metal ion inclusion was conducted by spin-coating ethanolic iron and nickel nitrate 0.8 wt % solutions on the polymer films. UVO treatment was employed to remove the polymer template as well as to convert the salt to a metal oxide. Samples were calcined in a tube furnace at 800 °C prior to etching to promote densification of the metal oxide and to remove residual polymeric material or convert the metal oxide film into a desirable state.^28^Figure 3 depicts the iron and nickel oxide nanoring arrays post UVO and calcination. The resultant patterns retain uniformity as the feature sizes of the BCP films and metal oxide masks closely follow normal distributions (Supporting Information, Figure S6). The micelle pattern is obvious from the metal oxide masks as only the PEO domain should incorporate the metal ion precursors. The outer nanoring diameters for iron and nickel oxide masks are 430 ± 113 and 423 ± 77 nm, respectively, and are both approx. 50 nm thick, which is favorable for sensing applications if feature spacing and size are optimized.^55,56^

**Figure 4:**
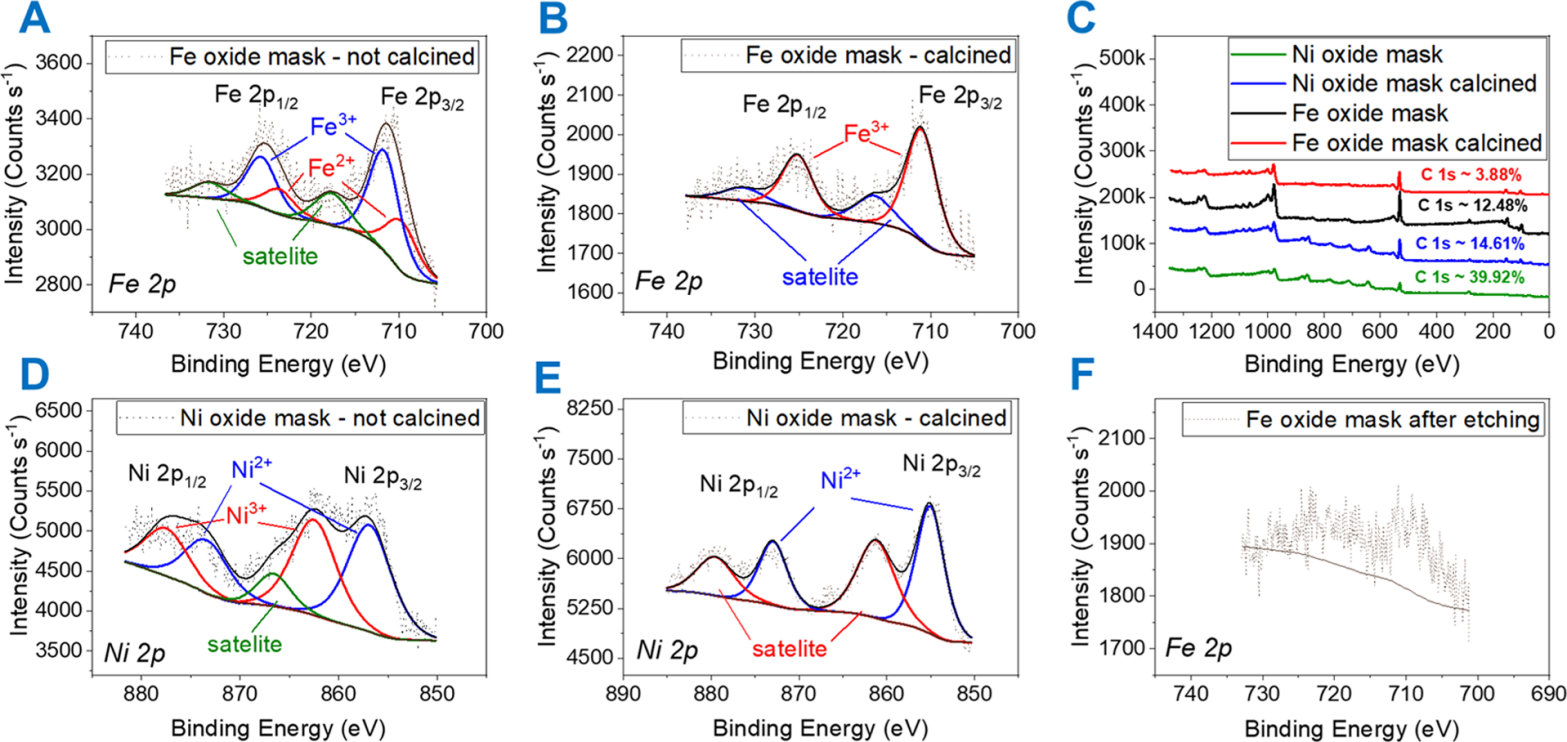
XPS spectra of metal oxide masks post UVO treatment: (A) iron oxide mask, (B) iron oxide mask after calcination, (C) comparison of survey spectra of the metal oxide mask and their relative carbon content, (D) nickel oxide mask, (E) nickel oxide mask after calcination, (F) iron oxide mask after ICP RIE (3 min) showing negligible iron content.

X-ray photoelectron spectroscopy (XPS) is essential to verify the chemical composition of the nanoring arrays produced from metal ion inclusion (Supporting Information, Figures S4 and 4). XPS spectra of metal oxide films were investigated to determine if the content of polymer or carbon had reduced upon calcination; it shows an approximate 3 times decrease in carbon content (Figure 4C). This verifies that UVO alone might not be enough to remove the polymer, which is undesirable if the nanoring array is purposed for optoelectronic applications. The chemical composition of the iron oxide is predominantly Fe_3_O_4_ upon UVO, which converts to Fe_2_O_3_ after calcination as reported by Ghoshal *et al.*(2) This is evident as an Fe 2p core level spectrum of iron oxide after UVO consists of broad Fe 2p_3/2_ (711.6 eV) and Fe 2p_1/2_ (725.4 eV) peaks, indicating the presence of both Fe^2+^ and Fe^3+^ ions (Figure 4A). The curve-fitted (Gaussian–Lorentzian) binding energies for Fe 2p_1/2_ and Fe 2p_3/2_ are 710.3 and 723.7 eV, attributed to Fe^2+^ and 711.9 and 725.8 eV assigned to Fe^3+^. The ratio of the curve-fitted peak areas Fe^3+^/Fe^2+^ is approximately 2 to 1, which is expected for Fe_3_O_4_. After calcination, the Fe 2p core level consists of sharper peaks: Fe 2p_3/2_ (711.0 eV), Fe 2p_1/2_ (725.1 eV), and high binding energy satellites, signifying the presence of only Fe^3+^ ions as anticipated for Fe_2_O_3_ as observed from Figure 4B. The nickel oxide mask consists of both Ni^2+^ and Ni^3+^ before calcination and upon annealing changes to NiO (Figure 4D,E).^54^

### Pattern Transfer

**Figure 5:**
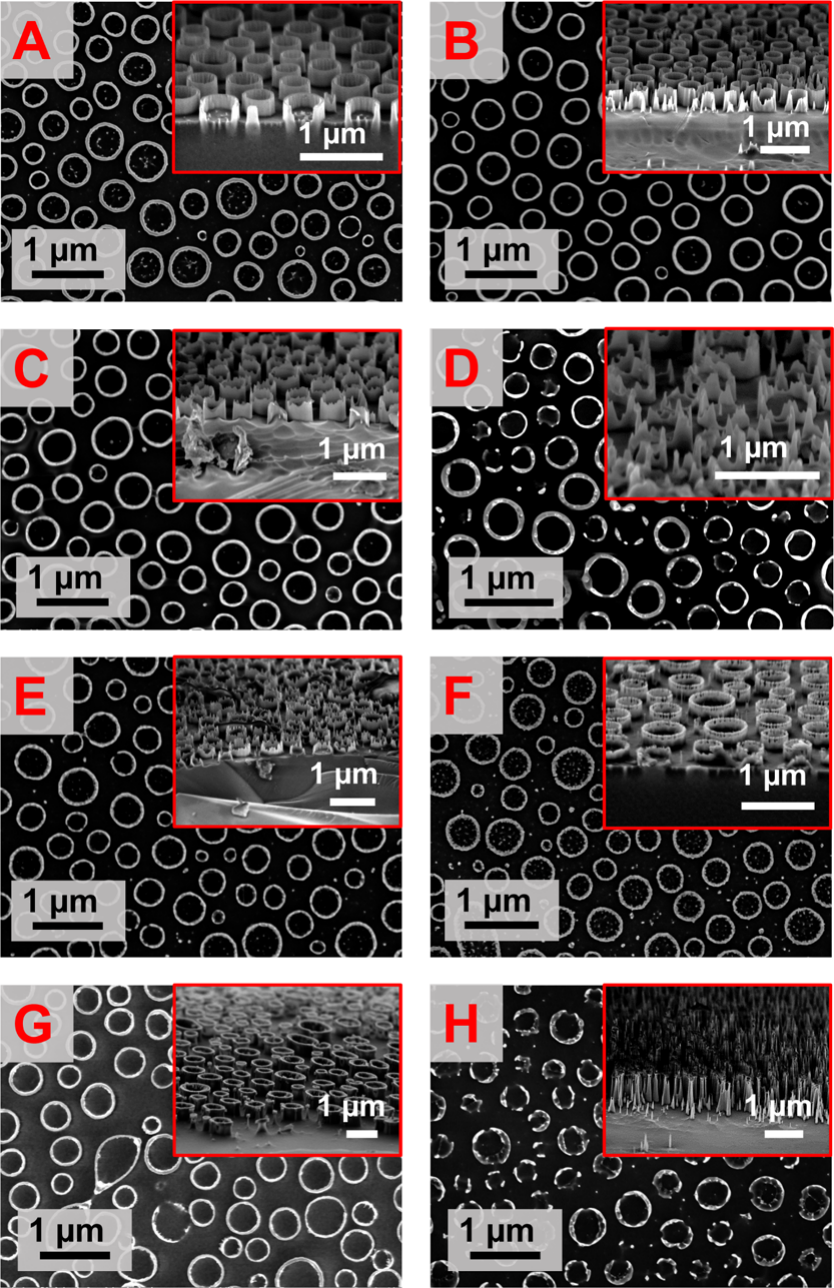
SEM micrographs (top-down and associated cross section at 70°) of patterned Si wafers. Morphology produced from a nickel oxide mask (not calcined) after: (A) 1 min etch, (B) 2 min etch, (C) 3 min etch, (D) 4 min etch. A pattern produced nickel oxide mask (calcined) after (E) 1 min etch, (F) 2 min etch, (G) 3 min etch, (H) 4 min etch.

In order to translate the metal oxide nanorings into 3D structures, we utilized a well-studied pattern transfer method.^53^ The ICP RIE method is outlined in the Experimental Section. We employed a CHF_3_–SF_6_ Si etch recipe without a native oxide etch step in order to preserve the integrity of the metal oxide mask during the aggressive etch. The advantage of this recipe is that the silicon tube walls are perpendicular to the substrate without the scalloping of the side walls commonly observed for other silicon ICP etch methods (see tilted cross-sectional scanning electron microscopy (SEM) insets in Figure 5).^18,53,57^ Nickel oxide masks produce better results after etching compared to iron oxide masks (Supporting Information, Figure S5). Therefore, the nickel oxide mask method was repeated for several etch times (1–4 min). Top-down and cross-sectional SEM micrographs in Figure 5 demonstrate defined large-scale silicon nanotube arrays post ICP etching. The resulting nanotube pattern after etching of the calcined nickel mask displays considerably lower feature heights as seen in Figure 5E–G as compared to the non-calcined mask in Figure 5A–C. This could be attributed to the lower stability of the calcined nickel oxide mask during this particular etch method, but more research is essential to verify whether the chemical composition of the nickel oxide mask gives rise to considerable differences during etching. For the non-calcined nickel oxide mask, etch times of 3 min or longer result in a lower feature height (209 ± 108 nm) than 1 or 2 min etch times (214 ± 24 and 355 ± 36 nm, respectively) due to nickel oxide mask removal during the ICP etch process. Furthermore, a 4 min etch leads to mask disintegration for both calcined and non-calcined masks and yields a disordered etched silicon film as seen from cross-sections in Figure 5D,H. The removal of the metal oxide mask during etching is supported by XPS in Figure 4F as the iron content after 3 min ICP RIE reduces considerably.

**Figure 6:**
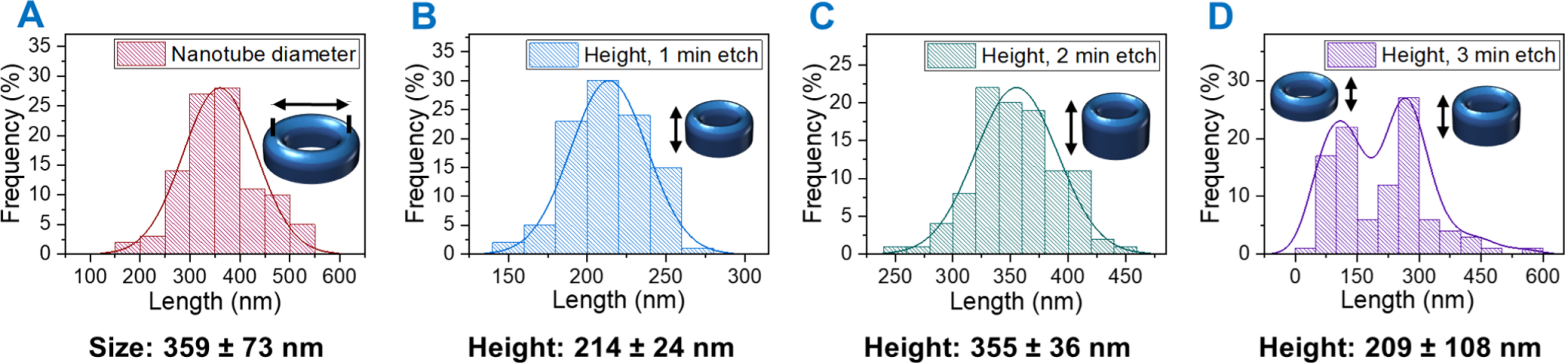
Size distributions of silicon nanotubes produced from the nickel oxide mask (not calcined): (A) nanotube diameter, (B) feature height after 1 min etch, (C) feature height after 2 min etch, (D) feature height after 3 min etch.

The feature sizes associated with the etched structures fabricated from nickel oxide nanorings (not calcined) are summarized in Figure 6. Overall, the feature sizes follow a normal distribution relatively well; however, the degree of uniformity remains considerably low. Nonetheless, we anticipate that a more ordered pattern may be yielded with further optimization and fine-tuning of the annealing conditions. Lower etch times of 1 or 2 min display a more uniform height distribution (Figure 6B,C), whereas longer times result not only in reduced height but also in a more irregular height distribution attributed to the onset of mask degradation (3 min etch in Figure 6D). Thus, the dry etch itself introduces more irregularity as the heights vary more with increasing etch time, signifying lack of control. We expect that further optimization of the etch recipe and gas chemistries may alleviate such issues. The nanotube array displays high porosity and a large surface area; the height of silicon nanotubes is 355 ± 36 nm after 2 min ICP RIE and the relative diameter distribution and wall thickness are the same as before the etch as seen in Figure 6A and Supporting Information Figure S6E–G. The nanotube diameter (359 ± 73 nm) and wall thickness (55 ± 11 nm) follow a normal distribution profile as shown in Figures 6A and S6G respectively. Therefore, we propose that the vertical silicon nanotube array described herein can find application in catalysis and sensing.^44,45,58^ The nanotube array itself can be modified, for example, as demonstrated by Fan and co-workers whereby an O_2_ environment at high temperatures is employed to oxidize Si to SiO_2_.^44^

## Conclusions

In this paper, we successfully obtained a silicon nanotube array using a PS-*b*-PEO (24–24.5 kg mol^–1^) toroidal micelle motif as a template for ICP RIE. The PS-*b*-PEO film readily self-assembled into toroidal micelles on a PS brush surface after spin coating. Additionally, film ordering was enhanced *via* WVA of the PEO block for an hour. The toroidal micelles displayed a size distribution of 400–600 nm, with an average micelle “pore” or inner diameter of 364 ± 101 nm. Although a notable variation in uniformity was observed, we suggest that this may be improved upon *via* co-casting the BCP with a PS homopolymer onto a PS brush substrate.^33,59,60^ Dynamic WVA could also be used to promote pattern uniformity across the film.^21^ Nevertheless, the method we employed in this work is a very attractive route to generate a relatively close-packed toroidal micelle array on the substrate.

Subsequently, we demonstrated the production of a metal oxide nanoring array (NiO and Fe_*x*_O_*y*_) by integrating metal ion precursors into the PEO corona of the toroidal micelle structure. We emphasize the versatility of this method as numerous different metals or moieties can be incorporated into the PEO domain with subsequent alterations such as calcining under different atmospheres to yield different metal state nanoring arrays.^61^ Various substrates can be employed for selective etching; however, for simplicity we chose a silicon substrate to show vertical Si nanotube fabrication.

The resulting metal oxide structures yielded an array of Si nanotubes with a feature diameter of 359 ± 73 nm, a thickness of 55 ± 11 nm, and a height of >300 nm after a 2 min ICP RIE process. Despite the clear success of this pattern transfer, we anticipate that even further improvement may be possible through additional refinement of the etch recipe.

Overall, we report a facile and scalable self-assembly method for generating arrays of Si nanotubes. The cost-effectiveness and time-efficiency of this strategy yield significant advantages over conventional top-down lithographic techniques, which we expect will find uses in materials and electronic applications such as transistors, sensors, and optoelectronic devices.^62^

## Experimental Section

### Materials

Hydroxy-terminated polystyrene (PS-OH) (number-average molecular weight, *M*_n_ = 10 kg mol^–1^, *M*_w_/*M*_n_ = 1.09, Mw: weight-average molecular weight), PS-*b*-PEO diblock copolymer (*M*_nPS_ = 24 kg mol^–1^, *M*_nPEO_ = 24.5 kg mol^–1^, *M*_w_/*M*_n_ = 1.09, *f*_PS_ = 0.49), PS (*M*_n_ = 16 kg mol^–1^, *M*_w_/*M*_n_ = 1.03), and PEG (*M*_n_ = 10 kg mol^–1^, *M*_w_/*M*_n_ = 1.01) were purchased from Polymer Source Inc. and were used without further purification. Silicon ⟨100⟩ p-type wafers with a native oxide layer were used as substrates. Fe_2_(NO_3_)3·9H_2_O (iron(III) nitrate nonahydrate), Ni(NO_3_)_3_·6H_2_O (nickel hydrate hexahydrate), toluene (CHROMASOLV, for HPLC, 99.9%), and ethanol (dehydrated, 200 proof) were purchased from Sigma-Aldrich and used without further purification unless otherwise stated. De-ionized water was used wherever necessary.

### Polymer Brush Preparation

Brush optimization is conducted according to the method reported by Lundy *et al.*(48,49) Silicon substrates were cleaned in toluene by sonication for 20 min and dried with N_2_ gas. Substrate surfaces were further cleaned, and hydroxyl-functionalized *via* oxygen plasma for 3 min (40 kHz, 120 W, Barrel Asher). The substrates were spin-coated (3000 rpm, 25 s, 5 s ramp) with 0.2 wt % PS-OH brush solution in toluene (stirred for 1 h). The polymer brush was annealed on a hot plate at 200 °C for 5 min. Subsequently substrates were sonicated in toluene for 20 min twice in order to remove any physiosorbed layers and dried in dry N_2_ gas afterward.

### BCP Film Preparation and Solvent Vapor Annealing

2 wt % solutions of PS-*b*-PEO BCP were prepared in toluene and stirred at 35 °C for 24 h. BCP solutions were spin-coated onto PS brush substrates at 3200 rpm for 25 s with a ramp of 5 s. WVA was conducted in a glass jar (150 mL) containing the sample and a small vial (50 mm × 12 mm × 4 mL) of water (3 mL) for 60 min at 25 °C. Samples were recovered from the jars and allowed to stand in air for the trapped solvent to evaporate.

### Metal Ion Inclusion

Metal inclusion and pattern transfer were conducted according to methods reported by various researchers.^1,18,27,63^ Fe_2_(NO_3_)_3_·9H_2_O and Ni(NO_3_)_3_·6H_2_O solutions of 0.8 wt % were prepared in ethanol. The metal ion solutions were spin-coated onto samples at 3200 rpm for 25 s with a 5 s ramp. UVO treatment for 3 h was employed to oxidize the precursor and remove the polymer *via* a UVO system (PSD Pro Series Digital UV Ozone System; Novascan Technologies, Inc., USA). Samples were then calcined in a tube furnace at 800 °C for 1 h in order to promote metal densification and remove cross-linked polymer residue.

### Pattern Transfer

Iron and nickel oxide were used as hard masks for pattern transfer *via* an Oxford Instruments Plasma Technology Plasmalab System100 ICP180 etch tool. Substrates were etched using an ICP power of 1200 W, RIE power of 20 W, etch time of 1 to 4 min, CHF_3_ (80 sccm), and SF_6_ (15 sccm) gases at 20 mTorr. Following pattern transfer, the remaining iron oxide and nickel oxide hard masks were removed *via* oxalic acid treatment.^53,64^

### Characterization

Atomic force microscopy (AFM) (Park systems, XE7) was operated in non-contact mode under ambient conditions using a silicon microcantilever probe tip (force constant of 42 N m^–1^). SEM (Zeiss Ultra Plus) images were recorded at an accelerating voltage of 2 kV and a working distance of 4–5 mm. SEM cross sections were obtained from samples cleaved in half and perpendicularly positioned to the incident electron beam with a tilt angle of 20° (70° to the incident beam). The film thicknesses were measured by reflectometry (Filmetrics F20) and electron microscopy.

TEM imaging and analysis were performed using an FEI Titian operating at 300 kV. Electron energy loss spectroscopy and energy-dispersive X-ray (EDX) analysis were performed using a Gatan Tridium EEL spectrometer and Edax EDX detector, respectively.

Dynamic CA measurements were recorded using a custom-built system on three different regions of each sample using a 60 Hz camera to capture the advancing CAs of three probe liquids (water, diiodomethane, and glycerol).^48,49^ The liquids were dispensed with a flow rate of 10 nL s^–1^ using a 35-gauge needle (Ø135 μm OD) with droplet volumes between 70 and 100 nL. SE was calculated using the advancing CAs *via* the Lifshitz–van der Waals/acid–base approach.^65^ CAs were measured on ImageJ using the “drop snake” plugin.^66,67^ SE was recorded for the BCP films and compared to the PS pellet and the PEG melt. The PS pellet was pressed according to Lundy *et al.*;^48^ PEG was directly melted onto Si wafers at 70 °C and tilted at 90° while cooling to produce a flat film.

XPS (VG Scientific ESCAlab Mk II) was performed under ultrahigh-vacuum conditions (<5 × 10^–10^ mbar) using a hemispherical analyzer and Al Kα X-rays (1486.6 eV).^48^ The emitted photoelectrons were collected at a take-off angle of 90° from the sample surface. The analyzer pass energies were set to 100 eV for survey scans and 40 eV for high-resolution core scans. Photoemission peak positions were corrected to C 1 s at a binding energy of 284.8 eV.^68^

(Where applicable, values are reported as sample mean value ± 1*s*, where *s* stands for sample standard deviation).

## Supporting Information Available

TEM characterization the brush; additional AFM, SEM, and XPS data; grafting density and SE calculations; and feature size distributions and reflectometry fits (PDF)
