# Supplementary material for: Large-Area Fabrication of Vertical Silicon Nanotube Arrays via Toroidal Micelle Self-Assembly
Source: Langmuir. 2021 Jan 28;37(5):1932–40. doi: 10.1021/acs.langmuir.0c03431 (PMC8280740; doi:10.1021/acs.langmuir.0c03431)
Supplement: Supplementary file 1 — la0c03431_si_001.pdf [file la0c03431_si_001.pdf]

Supporting Information

Large-Area Fabrication of Vertical Silicon Nanotube Arrays via Toroidal Micelle Self-Assembly

Nadezda Prochukhan,[a,b,*] Andrew Selkirk,[a] Ross Lundy,[a] Elsa C. Giraud,[a] Tandra Ghoshal,[a] Clive Downing,[a] Michael A. Morris[a,b,*]

[a] *School of Chemistry, CRANN and AMBER Research Centres, Trinity College Dublin, College Green, Dublin 2, Ireland.*

[b] *BiOrbic—Bioeconomy SFI Research Centre, University College Dublin, Belfield, Dublin 4, Ireland*

*Corresponding authors: prochukn@tcd.ie (N.P.)

morrism2@tcd.ie (M.A.M.)

Table of Contents

Section S1 – Additional PS brush and PS-*b*-PEO optimization data S2

Section S2 – Surface Energy measurements S3

Section S3 – XPS S4

Section S4 – Etch data S5

Section S5 – Feature sizes S6

Section S6 – Film Thickness data S7

References..S7

Section S1 – Additional PS brush and PS-*b*-PEO optimization data

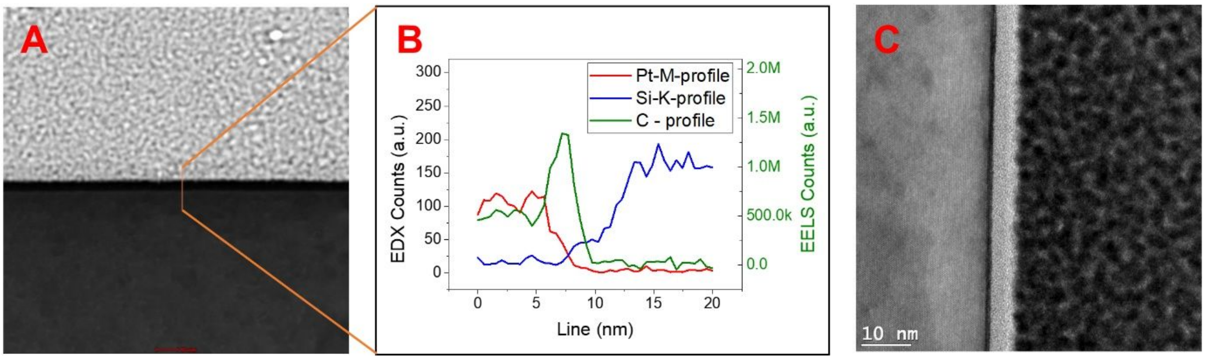

Figure S1. A. and **C.** TEM images of the PS-brush, thickness is approx. 5 nm. **B.** shows EDX and EELS profile of the line in **A**.

The rationale for using a PS brush: A physiosorbed PS layer can be used instead of a brush however a covalently grafted brush eliminates dewetting and provides a smoother surface by allowing washing to ensure single molecule polymer thickness. Root-mean square roughness (RMS roughness) of the brush ranges 0.2 to 0.8 nm, which is comparable to a clean Si surface (ca 0.2-0.5 nm) as estimated from AFM; therefore, it is assumed that roughness effects are negligible in pattern formation.

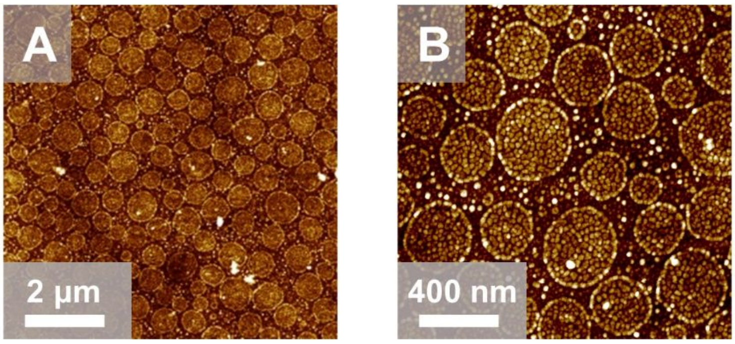

Figure S2. A. and **B.** 0.8 wt% water annealed PS-b-PEO film after Fe ion inclusion and UVO.

Grafting density σ of the PS brush is estimated as 0.32 chains per nm^2:[1]

$$\sigma = \frac{h_d \rho N_a}{M_n} \#[S1]$$

where h_d is the dry height of the brush (≈ 5 nm, estimated from TEM), ρ is the PS polymer density (1.05 g/cm^3), N_a is the Avogadro's number, and M_n is the number average molecular weight (≈ 10 kg mol^{-1}).

Section S2 – Surface Energy measurements

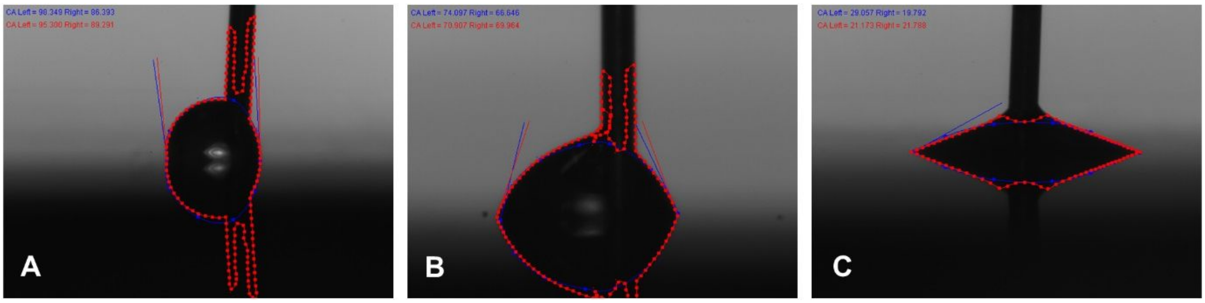

Figure S3. Representation of advancing contact angle (CA) measurements with drop-snake plugin on ImageJ for water annealed BCP sample for SE estimation. **A.** Water CA. **B.** Glycerol CA. **C.** Diiodomethane CA.

Surface energy was quantified from a 3 liquid system (water-glycerol-diiodomethane) *via* van Oss–Chaudhury–Good (vOCG) method.[2] Surface energy components of the liquids are described in **Table S1**.

Table S1. Surface Energy components for liquids used to calculate SE.

Liquid	Surface Energy Component			
	Total	Lifshitz–van der Waals (γ_{LW})	Lewis Acid (γ^{+})	Lewis Base (γ^{-})
**Water**	72.80	21.80	25.50	25.50
**Glycerol**	64.00	34.00	3.92	57.40
**Diiodomethane**	50.80	50.80	0.00	0.00

Surface energy was calculated by solving a system of linear equations:[2]

$$(\gamma_{S,LW}\gamma_{W,LW})^{0.5} + (\gamma_S^{+}\gamma_W^{-})^{0.5} + (\gamma_S^{-}\gamma_W^{+})^{0.5} = 0.5\gamma_W(1 + cos\theta_w) \#[S2]$$

$$(\gamma_{S,LW}\gamma_{G,LW})^{0.5} + (\gamma_S^{+}\gamma_G^{-})^{0.5} + (\gamma_S^{-}\gamma_G^{+})^{0.5} = 0.5\gamma_G(1 + cos\theta_G) \#[S3]$$

$$(\gamma_{S,LW}\gamma_{D,LW})^{0.5} + (\gamma_S^{+}\gamma_D^{-})^{0.5} + (\gamma_S^{-}\gamma_D^{+})^{0.5} = 0.5\gamma_D(1 + cos\theta_D) \#[S4]$$

$$\gamma_{S,AB} = 2(\gamma_S^{-}\gamma_W^{+})^{0.5} \#[S5]$$

$$\gamma_S = \gamma_{S,LW} + \gamma_{S,AB} \#[S6]$$

where γ are the SE components with subscripts S – solid, W – water, G – glycerol, D – diiodomethane, LW - Lifshitz–van der Waals, AB – acid-base and superscripts + and - signify acid and base respectively.

Section S3 – XPS

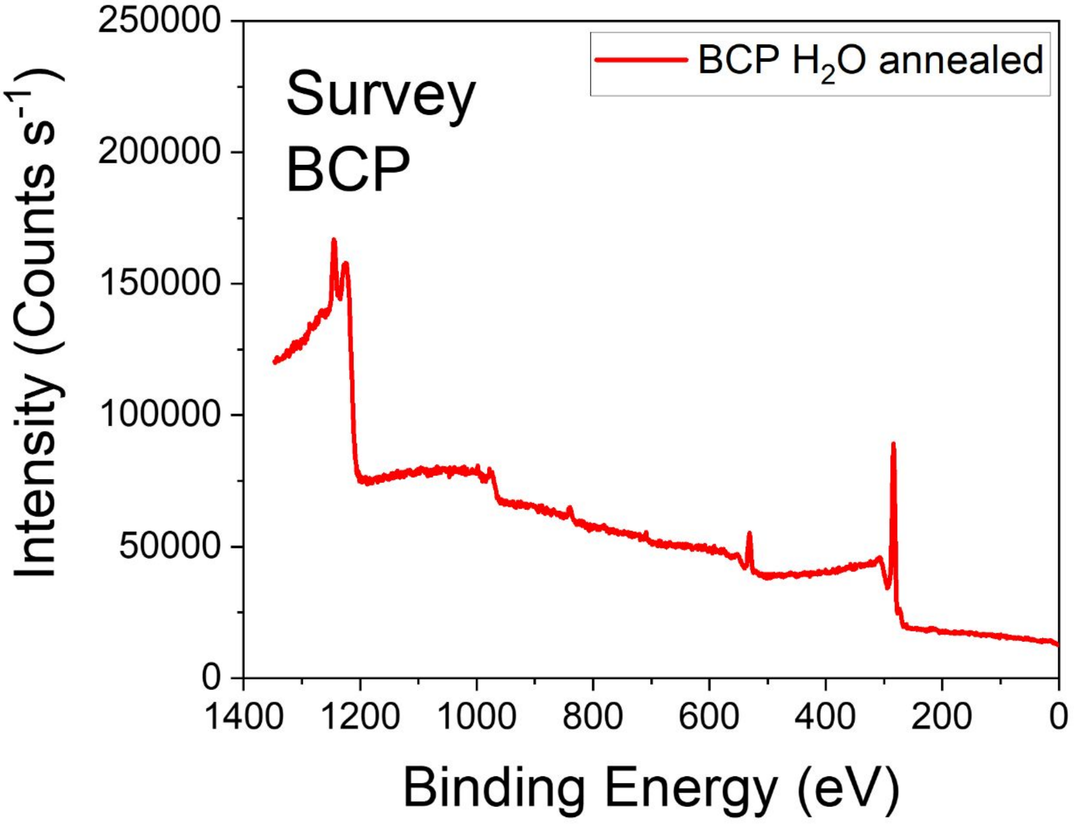

Figure S4. XPS survey spectra of BCP films.

Section S4 – Etch data

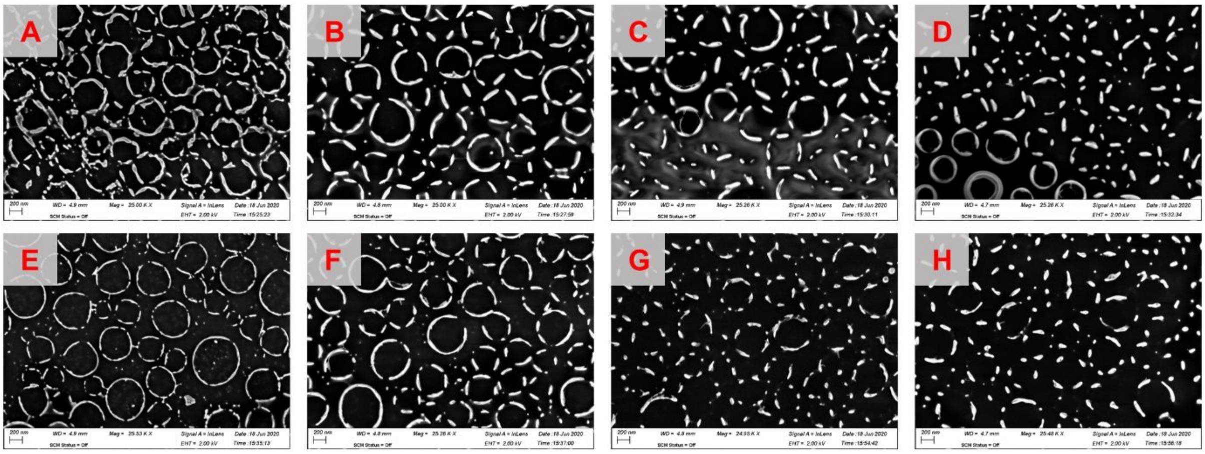

Figure S5. Etch pattern from H_2O annealed BCP Fe oxide mask (not calcined) after etching for **A.** 1 min, **B.** 2 min, **C.** 3 min, **D.** 4 min. Etch pattern from H_2O annealed BCP Fe oxide mask (calcined) after: **E.** 1 min, **F.** 2 min, **G.** 3 min, **H.** 4 min.

Section S5 – Feature sizes

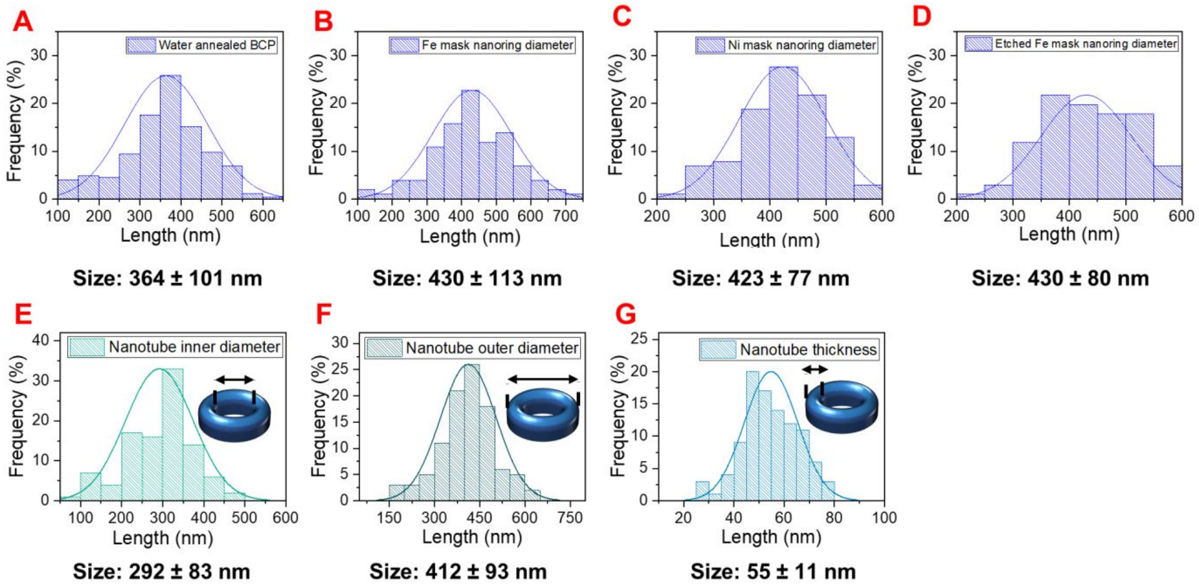

Figure S6. Feature sizes: "pore" diameters for PS-*b*-PEO water vapor annealed films and outer toroid diameter sizes for metal oxide masks: **A.** BCP film, **B.** Fe oxide mask, **C.** Ni oxide mask, and **D.** etched Fe oxide mask sample (1 min etch). After etching: **E.** nanotube inner diameter, **F.** nanotube outer diameter, **G.** nanotube thickness.

Section S6 – Film Thickness data

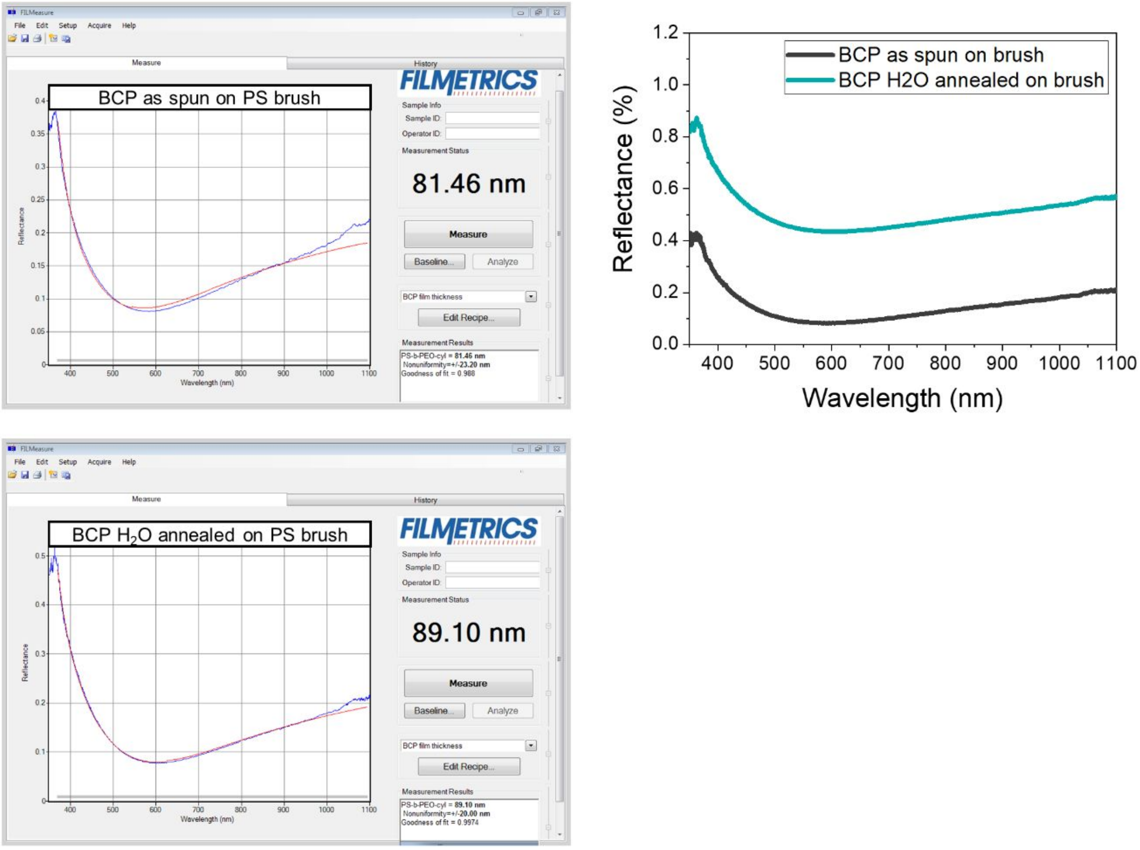

Figure S7. Examples of reflectometry fits for BCP films (labelled).

References

(1) Horst, R. J.; Brió Pérez, M.; Cohen, R.; Cirelli, M.; Dueñas Robles, P. S.; Elshof, M. G.; Andreski, A.; Hempenius, M. A.; Benes, N. E.; Damen, C.; De Beer, S. Swelling of Poly(Methyl Acrylate) Brushes in Acetone Vapor. *Langmuir* **2020**, *36* (40), 12053–12060. https://doi.org/10.1021/acs.langmuir.0c02510.

(2) Zenkiewicz, M. Comparative Study on the Surface Free Energy of a Solid Calculated by Different Methods. *Polym. Test.* **2007**, *26* (1), 14–19. https://doi.org/10.1016/j.polymertesting.2006.08.005.
